# ZnO nanoparticles as potential fertilizer and biostimulant for lettuce

**DOI:** 10.1016/j.heliyon.2022.e12787

**Published:** 2023-01-06

**Authors:** Carlos Alberto Garza-Alonso, Antonio Juárez-Maldonado, Susana González-Morales, Marcelino Cabrera-De la Fuente, Gregorio Cadenas-Pliego, América Berenice Morales-Díaz, Libia Iris Trejo-Téllez, Gonzalo Tortella, Adalberto Benavides-Mendoza

**Affiliations:** a Universidad Autónoma Agraria Antonio Narro, Doctorado en Ciencias en Agricultura Protegida, Calzada Antonio Narro, 1923, Buenavista, Saltillo, Coahuila, Mexico; b Universidad Autónoma Agraria Antonio Narro, Departamento de Botánica, Calzada Antonio Narro, 1923, Buenavista, Saltillo, Coahuila, Mexico; c CONACYT-UAAAN, Calzada Antonio Narro, 1923, Buenavista, Saltillo, Coahuila, Mexico; d Universidad Autónoma Agraria Antonio Narro, Departamento de Horticultura, Calzada Antonio Narro, 1923, Buenavista, Saltillo, Coahuila, Mexico; e Centro de Investigación en Química Aplicada, Enrique Reyna H. 140, San José de los Cerritos, Saltillo, Coahuila, Mexico; f Centro de Investigación y Estudios Avanzados del Instituto Politécnico Nacional Unidad Saltillo, Industrial Metalurgia, 1062, Parque Ind. Ramos Arizpe, Ramos Arizpe, Coahuila, Mexico; g Colegio de Postgraduados, Programa de Edafología, Carretera México-Texcoco km 36.5, Montecillo, Texcoco, Estado de México, Mexico; h Universidad de La Frontera, Centro de Excelencia en Investigación Biotecnológica Aplicada al Medio Ambiente, Francisco Salazar, 1145, Temuco, Chile

**Keywords:** Antioxidants, Plant nutrition, Plant physiology, Zinc, Protected agriculture, Nanofertilizers, Nanometal, Nanotechnology

## Abstract

Zn is an indispensable nutrient for crops that usually presents low bioavailability. Different techniques have been proposed to improve the bioavailability of Zn, including the use of nanofertilizers. The objective of the study was to evaluate the applications of drench (D) and foliar (F) ZnO nanoparticles (NZnO) compared to those of ionic Zn^2+^ (ZnSO_4_) in lettuce. The plants cv. Great Lakes 407 was produced in pots of 4 L with perlite-peat moss (1:1) under greenhouse conditions. The treatments consisted of NZnO applications that replaced the total Zn provided with a Steiner solution, as follows: Zn^2+^ (100%D) (control); Zn^2+^ (50%D+50%F); NZnO (100%D); NZnO (50%D+50%F); NZnO (75%D); NZnO (50%D); NZnO (75%F) and NZnO (50%F). Four applications of Zn were made with a frequency of 15 days. 75 days after transplant (DAP), the fresh and dry biomass, chlorophyll a, b, and β-carotene, phenolics, flavonoids, antioxidant capacity, vitamin C, glutathione, H_2_O_2_, total protein, and enzymatic activity of PAL, CAT, APX, and GPX were evaluated. The mineral concentrations (N, P, K, Ca, Mg, S, Cu, Fe, Mn, Mo, Zn, Ni, and Si) in the leaves and roots of plants were also determined. The results showed that, compared to Zn^2+^, NZnO promoted increases in biomass (14–52%), chlorophylls (32–69%), and antioxidant compounds such as phenolics, flavonoids, and vitamin C. The activity of enzymes like CAT and APX, as well as the foliar concentration of Ca, Mg, S, Fe, Mn, Zn, and Si increased with NZnO. A better response was found in the plants for most variables with foliar applications of NZnO equivalent to 50–75% of the total Zn^2+^ applied conventionally. These results demonstrate that total replacement of Zn^2+^ with NZnO is possible, promoting fertilizer efficiency and the nutraceutical quality of lettuce.

## 1 Introduction

The constant increment of the world's population leads us to be more efficient in producing food with better yields and high nutritional value. Zn is an essential element for plants; however, this element is deficient in the soils of various regions of the world. For the above, an appropriate supply of this element is considered vital to obtaining a higher yield and quality in agricultural crops [1]. Zn it is a component of some biomolecules (lipids and proteins), in addition to being a cofactor for auxins and playing an essential role in nucleic acids metabolism [2]. Additionally, this element is a component or activator of some enzymes, such as carbonic anhydrase (CA), alcohol dehydrogenase (ADH), and superoxide dismutase (SOD) [3]. Furthermore, Zn is involved in DNA transcription, RNA processing, and RNA editing in mitochondria and chloroplasts [4]. Zn is a component of cell membranes, participates in the expression and regulation of genes and biosynthesis of chlorophylls, in addition to participating in photosynthesis [5], mainly through the repair of protein D1 damaged by radiation during light harvesting in photosystem II [6].

Nanotechnology in agriculture is an alternative for increasing food production due to various applications, such as nanopesticides and nanofertilizers. The positive effects of nanomaterials (NMs) have been reported across multiple plant species, obtaining better responses in physiological processes, fruit quality, and yield [[7], [8], [9]]. However, NMs can also produce adverse effects, so the same material could produce biostimulation or toxicity [10] due to the size or shape of the NM, method of application, dose, exposure time, environmental conditions, and plant species [11].

Two main mechanisms have been identified in biostimulation by NMs. The first consists of the initial interactions of the NMs with the cell surface, inducing signals that trigger positive responses in plants. The second mechanism is due to the internalization of NMs, where their content becomes available for different metabolic functions of the plants [12]. The above modifies the metabolic process of plants, promoting vegetative growth and the production of antioxidant compounds, inducing greater tolerance or resistance to biotic-abiotic stresses [13].

NMs phytotoxicity (cytotoxicity, genotoxicity) is mainly due to time and levels of exposure of leaves, roots or seeds [14]. The primary mechanism by which some NMs produce genotoxicity is damage to chromosomes and interactions with DNA, causing plant mutations [15]. The interaction of NMs with cell walls and membranes induces cytotoxicity, causing oxidative stress [13], alterations in cell division, producing cells with malformations [16] and cell disorganization [17].

In addition to their biostimulant impact, NMs can be used as fertilizers, which have proven to have a higher efficiency than their conventional counterparts and a reduced environmental impact [18]. Mineral nutrition with NPs suggests greater efficiency than conventional fertilizer sources, which was demonstrated by applying nano-NPK at doses of 25 and 50% for the traditionally recommended values. As a result, higher yield, starch content, harvest index, and better efficiency in using nutrients in *Solanum tuberosum* cultivation were obtained [19]. Similarly, the application of nano NPK in chitosan formulations increased the content of N (17.04%), P (16.31%), and K (67.50%), in addition to promoting vegetative growth and chlorophyll content in *Coffea arabica* plants [20]. [21] reported the partial substitution of urea with urea-NPs in *Zea mexicana* plants, observing that the combination of both sources (50% conventional urea-50% urea NPs) showed higher levels of vegetative growth, in addition to increasing crude protein, carbohydrates, and detergent fiber.

In another study by Ref. [22]; it was found that application of hydroxyapatite-NPs to soil compared to the use of calcium superphosphate increased the leaf area and yield of *Brassica oleracea* var. Italic, while the foliar application of NPs of boron oxide vs. boric acid showed an increase in the same variables, in addition to a higher content of vitamin C. In both comparisons, the P and B contents were higher in plants with nanofertilizer applications. In another study [23], compared the application effects of Cu, Zn, Mn, and Fe in the NPs and ionic form on germination of lettuce, finding increases between 12 and 54% when nanofertilizers were used, demonstrating the potential of these agents in comparison with conventional microelement sources.

Recently, ZnO NPs (NZnO) were reported to increase antioxidant capacity and promote mineral absorption in *Cucumis sativus* [24]; however, the complete replacement of conventional fertilizers with Zn using nanoparticle formulations has not been reported. Nevertheless, this action could have significant benefits, such as the reduction of the use of conventional fertilizers, less environmental impact, and the obtaining of foods with better nutraceutical quality, in addition to facing the problems of deficiency or low availability of Zn in several regions of the world.

Based on all the above, the aim of this research was to compare different forms of application and levels of NZnO against ionic Zn (ZnSO_4_) on vegetative growth, photosynthetic pigments, bioactive compounds, and mineral concentrations in lettuce produced in a soilless system. We hypothesized that NZnO is as effective as Zn^2+^ as a plant nutrient and shows more efficiency.

## 2 Materials and methods

### 2.1 Establishment of the experiment

**Fig. 1:**
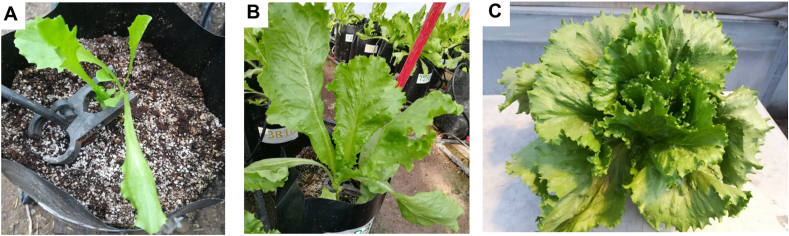
Lettuce plants used in the experiment. A: Transplant; B: Lettuce growth at 30 DAT; C: Plant at 75 DAT.

The experiment was established in the Department of Horticulture - Universidad Autónoma Agraria Antonio Narro (Saltillo, México). The plant material corresponds to the genotype “Great Lakes 407″ of KristenSeed, whose seeds had a germination percentage >85%. The seeds were germinated in expanded polystyrene trays with peat moss:perlite (1:1). After 28 days, the seedlings were transplanted in 4 L pots containing the same substrate mixture (Fig. 1-A). The substrate was subjected to a physicochemical analysis [25], where the presence of Zn was not detected.

Plant nutrition for the control treatment was supplied by a Steiner nutrient solution [26]. This solution was prepared at a concentration of 50% and constant pH of 6.5, containing the following quantities of each element (in mg L^−1^): N: 131; P: 31; K: 274; Ca: 168; Mg: 49; S: 133; B: 0.43; Fe: 3.2; Cu: 0.02; Mn: 1.94; Zn: 0.0227; Mo: 0.01, which was supplied as irrigation water, applying 1 L plant day^−1^. Plants assigned to NZnO treatments received Steiner solution without Zn^2+^. The water used for irrigation was chemically analyzed without detecting Zn concentrations. The plants were kept for 75 days after transplant (DAT) (Fig. 1-B and C) in a chapel-like greenhouse with homogeneous conditions of temperature (25–27 °C) and relative humidity (60–70%).

### 2.2 ZnO NPs and applied treatments

**Fig. 2:**
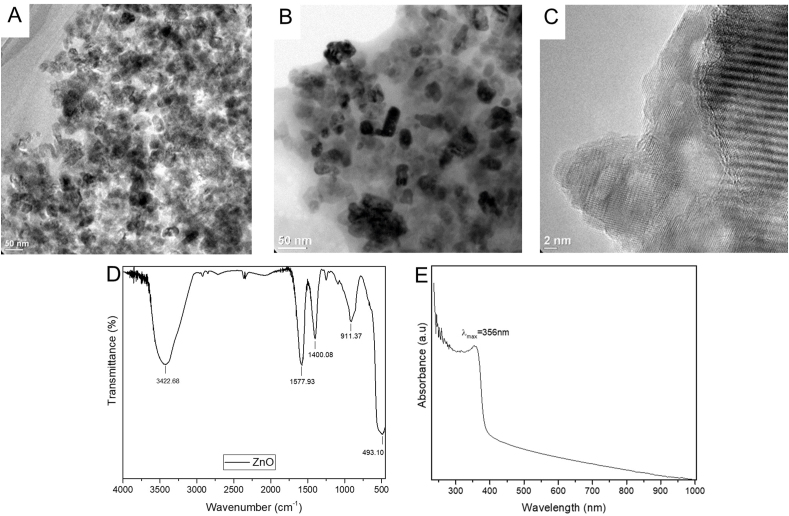
TEM (A–B) and HRTEM (C) images. FTIR (D) and UV–Vis (E) spectra of ZnO nanoparticles. Subfigures A-C from Ref. [28].

ZnO NPs were synthesized based on the methodology of [27]. A complete description of the synthesis method was previously reported [28]. The morphology and structure of NPs were analyzed by transmission electron microscopy (TEM) and high-resolution transmission electron microscopy (HRTEM), where most NPs resulted in a quasi-spherical shape (Fig. 2-A [28]), an average diameter of 16.49 nm (Fig. 2-B), and crystalline appearance (Fig. 2-C). Additionally, Fourier Transform Infrared Spectroscopy (FTIR) and UV–Vis tests were performed. The FTIR spectrum (Fig. 2-D) showed a strong peak between 493.1 cm^−1^, corresponding to the stretching vibrations of ZnO bands, which indicates that the samples are well crystallized [29]. The UV–Vis spectrum illustrated in Fig. 2-E shows an adsorption peak located at 356 nm, which is attributed to the intrinsic band gap of ZnO absorption. Similar values of the absorption band that represent ZnO NPs was also reported in previous works in which the range of the absorption band was from 355 to 380 nm [30].

Treatments consisted of foliar applications (F) and drench (D) of different levels of NZnO, compared to applications of conventional Zn^2+^ (ZnSO_4_). The application of Zn^2+^ at a concentration of 0.227 mg L^−1^ was considered the control because Zn^2+^ is the main supply for this element in soilless production systems. The total amount of Zn^2+^ calculated for a lettuce crop (17 mg Zn plant^−1^ in 75 days) was taken as the basis for the applications of the treatments, which consisted of the two forms of application and percentages of Zn with respect to the control, as follows:

T1: Zn^2+^ (100%D) (control), equivalent to 17 mg Zn plant^−1^.

T2: Zn^2+^ (50%D+50%F), equivalent to 17 mg Zn plant^−1^.

T3: NZnO (100%D), equivalent to 17 mg Zn plant^−1^.

T4: NZnO (50%D+50%F), equivalent to 17 mg Zn plant^−1^.

T5: NZnO (75%D), equivalent to 12.75 mg Zn plant^−1^.

T6: NZnO (50%D), equivalent to 8.5 mg Zn plant^−1^.

T7: NZnO (75%F), equivalent to 12.75 mg Zn plant^−1^.

T8: NZnO (50%F), equivalent to 8.5 mg Zn plant^−1^.

The total number of applications of Zn was four (4.25 mg Zn^−1^ each), with an interval of 15 days between each.

### 2.3 Evaluated variables

#### 2.3.1 Fresh-dry biomass of plants

After 75 DAT, two lettuce plants from each experimental unit were harvested and separated into leaves and roots to determine fresh biomass. Subsequently, the most recently mature leaves and a portion of the roots were taken and washed with distilled water and later placed in a freezer at −20 °C for future analyses. The remaining plant material was dehydrated in a drying oven at 65 °C for 72 h to determine dry biomass.

#### 2.3.2 Sample processing for biochemical analysis

The previously frozen leaf and root samples were lyophilized to avoid the denaturation of biochemical compounds. This process was carried out in a liophyllizer model Labconco FreeZone 4.5 (Labconco Inc., Kansas City, USA) at a temperature from −45 °C during seven days. Subsequently, the tissue was macerated for further analysis. This lyophilized tissue (LT) was used for all photosynthetic pigments, bioactive compounds, and enzymatic activity analyses.

#### 2.3.3 Photosynthetic pigments

The concentrations of chlorophyll a (Chla), b (Chlb), total (Chla + b), and β-carotene (β-car) were analyzed in lyophilized leaves. A mix of 10 mg of LT + 2 mL of hexane:acetone (3:2) was centrifuged (12000 rpm, 10 min, 4 °C). The resulted extract was read in a spectrophotometer model Unico UV2150 (Unico Inc., New Jersey, USA) at different wavelengths (505, 453, 645, and 663 nm). The resulted absorbances was used for later calculation with equations proposed by Ref. [31]; expressing the results in mg 100 g^−1^ DW. Subsequently, the Chla/Chlb and Chla + b/β-car ratios were calculated.

#### 2.3.4 Bioactive compounds and enzymatic activity

The concentration of total phenolics was obtained using a Folin-Ciocalteu reaction. First, 100 mg of LT+1 mL of water:acetone (1:1) was mixed and centrifuged (12500 rpm, 10 min, 4 °C). Subsequently, 50 μL of resulted supernatant +200 μL of reagent Folin Ciocalteu +0.5 mL of Na_2_CO_3_ (20%) + 5 mL of H_2_O were homogenized and placed for 30 min in a water bath (45 °C). After this time, the sample was read at 750 nm in a spectrophotometer model Thermo Fisher G10S (Thermo Fisher Scientific, Massachusetts, USA). The same equipment was used for the readings of flavonoids, vitamin C, glutathione, H_2_O_2_, and antioxidant enzymes. The concentration of total phenolics was reported as mg 100 g^−1^ DW. All the above following the method described by Ref. [32].

The concentration of total flavonoids was determined mixing 100 mg of LT + 10 mL of methanol and subsequently filtered with a Whatman Filter (No 1001). Later, a mix of 2 mL of solution and 2 mL of AlCl_3_ (2%) was incubated in dark conditions for 20 min. After this time, the sample was read at 415 nm, reporting the results as mg 100 g^−1^ DW. All the above following the techniques described by Ref. [33].

Vitamin C concentration was obtained with a mix of 10 mg of LT + 1 mL of HPO_3_ (0.36 M), which later was centrifuged (5000 rpm, 10 min, 4 °C). After this, a mix of 200 μL of supernatant +1.8 mL of 2,6-diclorfenolindofenol (2,6 D-0.09 M) was read at 515 nm, expressing the results as mg g^−1^ DW. All the above according with [34].

Glutathione (GSH) was quantified with a mix of 100 mg of LT + 1.5 mL of phosphate buffer (K_2_HPO_4_ 0.01 M + KH_2_HPO_4_ 0.01 M) (1:1) + 10 mg of polyvinylpyrrolidone (PVP), which later was centrifuged (12500 rpm, 10 min, 4 °C) and subsequently filtered using filters of nylon membrane (0.45 μm). This extract was used for the quantification of GSH, antioxidant capacity, protein, and activity of antioxidant enzymes. The GSH concentration was determined using a mix of 480 μL of extract +320 μL of DTNB reagent (1 mM) + 2.2 mL of Na_2_HPO_4_ (0.32 M), which later was read at 412 nm, reporting the results as mmol 100 g^−1^ DW. All the above following the techniques described by Ref. [35].

Antioxidant capacity was obtained using 6 μL of extract +254 μL of DPPH radical (2,2-diphenyl-1-picrylhydrazyl, 6.34 M). This mix was placed and read at 630 nm in a microplate reader model BioTek Elx808 (BioTek Inc., Vermont, USA), expressing the results as μmol g^−1^ DW, according with [36].

The concentration of H_2_O_2_ was quantified by the extraction of 10 mg of LT + 1 mL of trichloroacetic acid (0.1%), which latter was centrifuged (12000 rpm, 15 min, 4 °C). Subsequently, a mix of 500 μL of supernatant +750 μL of phosphate buffer (KH_2_HPO_4_ 0.01 M + K_2_HPO_4_ 0.01 M, 1:1) was read at 390 nm, expressing the results as μmol g^−1^ DW. All the above following the methods proposed by Ref. [37].

The concentration of total protein (TP) was quantified with a mix of 5 μL of the extract + 250 μL Bradford's reagent, which was incubated for 10 min in dark conditions. After this, the samples were read in a microplate reader at 630 nm. The results were reported as mg g^−1^ DW. All the above according with [38]. These TP values were used to calculate the enzymatic activity.

The activity of catalase (CAT) (EC 1.11.1.6) was quantified with a mix of 100 μL of the extract + 1 mL of H_2_O_2_ (100 mM) + 400 μL of H_2_SO_4_ (5%), which was directly read at 270 nm. Subsequently, a second lecture was taken after 1 min, with the objective of calculate the activity of CAT in 1 min of reaction. This activity was expressed as U g^−1^ TP, (U: mM equivalents of H_2_O_2_ consumed mL^−1^ min^−1^). All the above according with [39].

Ascorbate peroxidase (APX) (EC 1.11.1.11) was determined with a mix of 100 μL of the extract + 1 mL of H_2_O_2_ (100 mM) + 500 μL of ascorbate + 400 μL of H_2_SO_4_ (5%), which was directly read at 266 nm. Subsequently, a second lecture was taken after 1 min, with the objective of calculate the activity of APX in 1 min of reaction. This activity was expressed as U g^−1^ TP, (U: μmol of oxidized ascorbate mL^−1^ min^−1^). All the above following the methodology proposed by Ref. [40].

Phenylalanine ammonium lyase (PAL) (EC 4.3.1.5) was obtained with a mix of 100 μL of the extract + 900 μL of phenylalanine (6 mM), which was placed in water bath (40 °C, 30 min). Subsequently, were added 250 μL of HCL (5 N) + 750 μL of H_2_O and directly read at 290 nm. The PAL activity was expressed as U g^−1^ TP, (U: μM of trans-cinnamic acid mL^−1^ min^−1^). All the above according with [41].

Glutathione peroxidase (GPX) (EC 1.11.1.9) was determined through a mixture of 200 μL of the extract + 200 μL of Na_2_HPO_4_ (0.067 M) + 400 μL of GSH (0.01 M), which was placed in water bath (25 °C, 5 min). After this time, 200 μL of H_2_O_2_ (1.3 mM) was added. Ten minutes later, one milliliter of trichloroacetic acid (1%) was added and centrifuged (3000 rpm, 10 min, 4 °C). Finally, a mix of 480 μL of resulted supernatant +2.2 mL of Na_2_HPO_4_ (0.32 M) + 320 μL of DTNB (1 mM) was read at 412 nm. The GPX activity was reported as U g^−1^ TP, (U: mM glutathione equivalents reduced mL^−1^ min^−1^). All the above according with [42].

#### 2.3.5 Leaf-root mineral concentration

The determination of N was carried out by the micro-Kjeldahl method following the methodology of [43]. To quantify the concentrations of P, K, Ca, Mg, S, Cu, Fe, Mn, Mo, Zn, Ni, and Si, the previously dried samples were first subjected to acid digestion with HNO_3_. Then, the extract obtained was read in a coupled plasma emission spectrophotometer (ICP-OES) model Optima 8300 (PerkinElmer, MA, USA) according to Ref. [44]. All results were reported as mg kg^−1^ DW.

### 2.4 Statistical analysis

The experiment was established under a completely randomized experimental design with eight treatments and five repetitions, obtaining 40 experimental units with two plants as experimental units. The Levene and Kolmogorov–Smirnov tests were previously carried out to verify the equality of variances and normal distribution of the data. The data were analyzed through an analysis of variance (ANOVA), and a Fisher's least significant difference (LSD) mean comparison test was made for variables with a p ≤ 0.05. Additionally, a correlation analysis was made between all the variables evaluated. All the statistical analysis were performed in the Infostat v. 2020 software.

## 3 Results

### 3.1 Fresh-dry biomass of plants

The application of NZnO favored the gain of fresh and dry matter in lettuce plants. The most significant increase in fresh leaf biomass was found in the NZnO (75%) treatment, which was 52% higher than that in the control. Next were treatments of NZnO (50%D+50%F), Zn^2+^ (50%D+50%F), and NZnO (50%D), with increases over the control of 25, 23, and 21%, respectively. The NZnO (100%D) treatment showed an increase over the control of 14%. A similar response was found in dry weight of the leaves. The NZnO (75%) treatment favored a 39% increase over the control, followed by NZnO (50%D) treatment with 29%, and NZnO (100%D) and NZnO (50%D+50%F), both with increases of 27%.

In contrast, there was a decrease in fresh root biomass, with the NZnO (75%D) and NZnO (50%F) treatments showing reductions of 38% and 20%, respectively, in comparation of control. Similarly, the dry biomass of the roots was negatively affected by the NZnO (75%D) treatment, with a decrease of 51% compared to the control, while the NZnO (75% F), NZnO (100%D), and NZnO (50%D) treatments reduced the values of this variable by 31, 24 and 23%, respectively (Table 1).

**Table 1:** Fresh and dried biomass in leaf and lettuce root.

TREATMENT	LFW (g)		LDW (g)		RFW (g)		RDW (g)	
Zn^2+^(100%D)	402.88	e	18.94	c	51.14	ab	7.02	a
Zn^2+^(50%D+50%F)	498.34	bc	22.26	bc	58.16	a	6.04	ab
NZnO (100%D)	461.52	cd	24.04	ab	53	ab	5.36	b
NZnO (50%D+50%F)	506.1	b	23.98	ab	49.9	b	6.83	a
NZnO (75%D)	407.16	e	21.62	bc	31.88	d	3.42	c
NZnO (50%D)	489.38	bc	24.54	ab	45.96	bc	5.38	b
NZnO (75%F)	612.72	a	26.34	a	50.72	ab	4.87	b
NZnO (50%F)	428.4	de	21.6	bc	41.36	c	5.88	ab

LFW: Leaf fresh weight; LDW: Leaf dry weight; RFW: Root fresh weight; RDW: Root dry weight. Values are the mean of treatments. Different letters in each column indicate a significant difference (LSD, *p* ≤ 0.05). n = 5.

### 3.2 Photosynthetic pigments

**Fig. 3:**
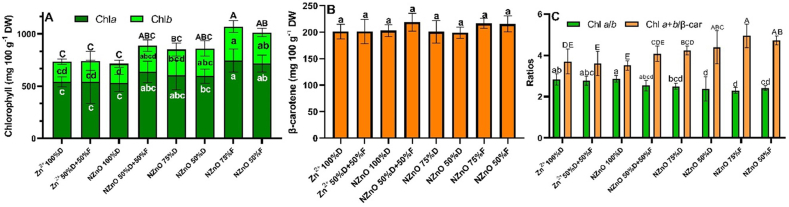
Photosynthetic pigments in lettuce leaves. A: Chl*a*, Chl*b*, and Chl*a + b*. Letters inside bars are the mean comparation for each chlorophyll type. Capital letters on the bars are mean comparation of Chl*a + b*; B: β-carotene; C: Chl*a*/*b* and chl*a* + *b*/β-carotene ratios. Lowercase letters are mean comparation of Chl*a*/*b,* and capital letters are mean comparation of chl*a* + *b*/β-carotene ratio. Different letters indicate a significant difference (LSD, *p* ≤ 0.05). The lines on the bars indicate the standard error of the mean. n = 5.

Chlorophylls in leaves increased with NZnO applications. Chla showed an increase with foliar application of NZnO, while the NZnO (75%F) and NZnO (50%F) treatments registered increases of 37% and 32%, with respect to control. In addition, Chlb resulted in a 69% increase for the NZnO (75%F) treatment and a 54% increase for the NZnO (50%F) treatment. The same trend was observed in the Chla + b concentration, with increases of 45 and 38% for the NZnO (75%F) and NZnO (50%F) treatments, respectively (Fig. 3-A). On the other hand, the treatments did not affect the concentration of β-car (Fig. 3-B).

The Chla/Chlb ratio, it resulted in a slight decrease in the NZnO (50%F) treatment, with a ratio 15% lower compared to the control, while in NZnO (50%D) and NZnO (75%F), decreases of 16% and 19% were observed. In contrast, compared with the control, the Chla + b/β-car ratio increased by 19% in plants with NZnO (50%D) and showed 28 and 33% increases with the NZnO (50%F) and NZnO (75%F) treatments, respectively (Fig. 3-C).

### 3.3 Bioactive compounds and enzymatic activity

**Fig. 4:**
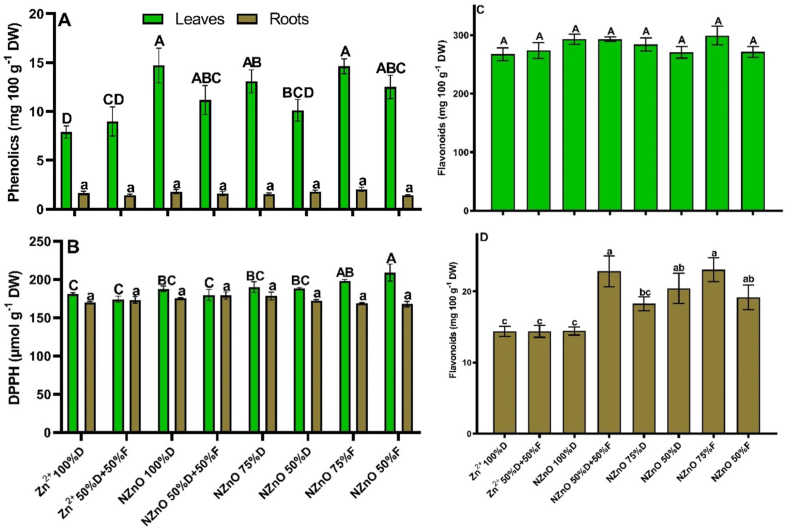
Phenolics (A), antioxidant capacity DPPH (B), leaf flavonoids (C), and root flavonoids (D) in lettuce. Capital letters on the bars are the mean comparison of the leaves. Lowercase letters on the bars are the mean comparation of roots. Different letters on the bars indicate a significant difference (LSD, *p* ≤ 0.05). The lines on the bars indicate the standard error of the mean. n = 5.

The phenolic compounds in leaves resulted in increases when making NZnO applications. The highest increase was found in plants of the NZnO (100%D) treatment, which promoted the concentration of these compounds by 86%, followed by NZnO (75%F), NZnO (75%D), and NZnO (50%F), with increases of 85, 65, and 58%, respectively (Fig. 4-A). This compounds in lettuce roots were not altered by the application of Zn^2+^ or NZnO (Fig. 4-A). Similarly, the antioxidant capacity (DPPH) of the leaves resulted in increases of 15 and 9% compared to the control when applying NZnO (50%F) and NZnO (75%F), respectively. There were no differences in DPPH of the roots between treatments (Fig. 4-B). Regarding the concentration of flavonoids, no difference was found in the leaves (Fig. 4-C). However, in the case of the roots, the application of NZnO promoted an increase in these compounds, mainly in the NZnO (75%F) treatment, where a rise of 60% was observed with respect to control, followed by the NZnO (50% D+50%F), NZnO (50%D), and NZnO (50%F) treatments, with increases of 58, 42, and 33%, respectively (Fig. 4-D).

**Fig. 5:**
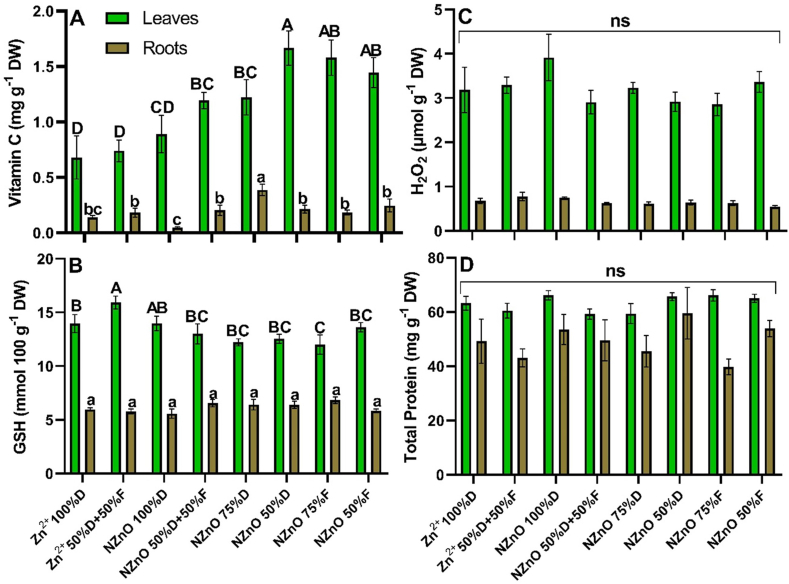
Vitamin C (A), GSH (B), H_2_O_2_ (C), and total protein (D) in leaves and roots of lettuce. Capital letters on the bars are the mean comparison of the leaves. Lowercase letters on the bars are the mean comparation of roots. Different letters on the bars indicate a significant difference (LSD, *p* ≤ 0.05). ns = no significant difference. The lines on the bars indicate the standard error of the mean. n = 5.

The vitamin C concentration increased with most NZnO treatments, where NZnO (50%D) showed a 145% increase over the control, followed by NZnO (75%F) and NZnO (50%F) treatments, with increases of 132% and 111%, respectively, while NZnO (75%D) and NZnO (50%D+50%F) treatments recorded 79% and 75% increases in vitamin C concentration (Fig. 5-A). In the case of the roots, the NZnO treatment (75%D) showed a rise of 178% in vitamin C concentration compared (Fig. 5-A). The concentration of GSH in leaves showed a 14% increase in the Zn^2+^ (50%D+50%F) treatment and a contrasting 15% negative effect for the NZnO (75%F) treatment. On the other hand, the concentration of GSH in roots was not altered (Fig. 5-B). The treatments did not alter the concentration of H_2_O_2_ in lettuce leaves and roots (Fig. 5-C) or in the case of total protein in either vegetative organ (Fig. 5-D).

**Fig. 6:**
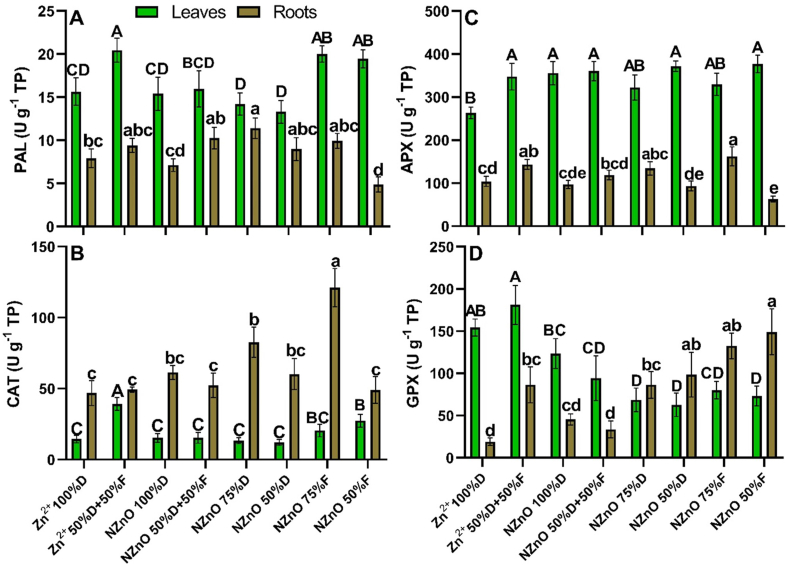
PAL (A), CAT (B), APX (C), and GPX (D) activities in leaves and roots of lettuce. Capital letters on the bars are the mean comparison of the leaves. Lowercase letters on the bars are the mean comparation of roots. Different letters on the bars indicate a significant difference (LSD, *p* ≤ 0.05). The lines on the bars indicate the standard error of the mean. n = 5.

The enzymatic activity was positively or negatively affected by the applied treatments. Compared to the control, PAL activity in leaves increased 31% for the Zn^2+^ (50%D+50%F) treatment and 28% and 24% for the NZnO (75%F) and NZnO (50%F) treatments, respectively (Fig. 5-A). On the other hand, PAL activity in lettuce roots increased by 44% with the application of NZnO (75%D) but was reduced by 38% with NZnO (50%F) (Fig. 6-A). Regarding CAT activity in leaves, the Zn^2+^ (50%D+50%F) treatment promoted the activity by 170%, followed by the NZnO (50%F) treatment, which increased the activity of this enzyme by 88% compared to the control (Fig. 6-B). On the other hand, the activity of CAT in roots showed an increase of 158% with the application of the NZnO (75%F) treatment, followed by the NZnO (75%D) treatment, which promoted enzymatic activity by 78% in comparison with the control (Fig. 6-B).

On the other hand, the activity of APX in leaves increased with most treatments, being as follows: NZnO (50%F) > NZnO (50%D) > NZnO (50% D+50%F) > NZnO (100%D) > Zn^2+^ (50% D+50%F), where the increases were 43, 41, 36, 35 and 31%, respectively (Fig. 6-C). In the case of the roots, the activity of APX was only increased in plants of the NZnO (75%F) and Zn^2+^ (50% D+50%F) treatments, which showed increases of 56% and 38%, respectively, while the application of the NZnO (50%F) treatment produced a 40% reduction in the activity of this enzyme (Fig. 6-C).

Unlike the enzymes mentioned above, GPX activity in leaves was adversely affected by NPS treatments, being as follows: NZnO (50%D)> NZnO (75%D)> NZnO (50%F)> NZnO (75%F)> NZnO (50%D+50%F), with reductions of 60, 56, 53, 49 and 39%, respectively, compared to control treatment (Fig. 6-D). In contrast, the activity of this enzyme was considerably increased in the roots, where the most significant increase was found in the NZnO (50%F) treatment, with 681% higher activity compared to the control, followed by the NZnO (75%F), NZnO (50%D), and NZnO (75%D) treatments, with increases over the control of 594, 416 and 353%, respectively. Likewise, the Zn^2+^ (50%D+50%F) treatment showed a 350% of increase in activity of GPX compared to the control (Fig. 6-D).

### 3.4 Leaf-root mineral concentration

The application of NZnO significantly altered the concentration of minerals in lettuce leaves and roots. In the case of N, the ANOVA did not show a significant difference between the treatments in either leaves or roots. However, in the concentration of P in leaves, a reduction was found for most NZnO treatments, with the most significant decrease in NZnO (75%D) treatment with a decline of 41% compared to control, followed by NZnO (50%D), NZnO (50%D+50%F), NZnO (100%D) and NZnO (75%F) treatments with decreases of 36, 35, 28, and 27%, respectively, finding no difference between the Zn^2+^ (50%D+50%F) treatment and the control.

A similar trend was found in the roots, in which the P decreased in all treatments with the application of NZnO, as follows: NZnO (50%D) > NZnO (75%D) > NZnO (50%D+50%F) > NZnO (50%F) > NZnO (100%D) > NZnO (75%F), with decreases of 65, 54, 53, 45, 35 and 29%, respectively, compared to the control; Zn^2+^ (50%D+50%F) treatment reduced the P concentration by 30%. In the case of K in leaves, only a 33% reduction was found in the NZnO (50%F) treatment compared to the control. However, the concentration of this element in the roots was negatively affected by NZnO applications, with a 62% reduction in the NZnO (50%D) treatment, followed by the NZnO (75%D), NZnO (50%D+50%F) and NZnO (50%F) treatments with reductions of 59, 58, and 36%, respectively.

On the other hand, the concentrations of Ca, Mg, and S in the leaves increased with NZnO applications. Ca increased by 71% and 66% for the NZnO (100%D) and NZnO (50%D+50%F) treatments, respectively. The Mg levels increased in the NZnO (50%F) and NZnO (50%D+50%F) treatments by 39% and 34%, respectively. For S, a similar trend was obtained, with increases of 30, 23, and 21% in the NZnO (50%F), NZnO (100%D), and NZnO (75% F) treatments, respectively. In contrast, the concentrations of Ca and Mg in the roots decreased when NZnO was applied. Ca was reduced in most treatments, remaining as follows: Zn (50%D)> NZnO(50%D+50%F)> NZnO(75%F)> NZnO(50%F)> NZnO(100%D), with decreases relative to the control of 37, 30, 28, 26, and 17%, respectively, and a decrease of 39% in plants of Zn^2+^ (50%D+50%F) treatment. A similar effect was found for Mg, for which the NZnO (75% F) and NZnO (50%D) treatments reduced the concentration of this element in the roots by 28 and 24%, respectively. For its part, the Zn^2+^ (50%D+50%F) treatment produced a Mg decrease of 39%. The concentration of S in the roots was not modified by NZnO applications (Table 2).

**Table 2:** Concentration of macronutrients (mg kg^−1^ DW) in leaves and roots of lettuce.

TREATMENT	N		P		K		Ca		Mg		S	
LEAVES												
Zn^2+^(100%D)	26310	ab	3143.13	a	35312.5	abc	14553.13	cd	2906.25	c	1935	c
Zn^2+^(50%D+50%F)	27920	a	3194.06	a	29825	bcd	18253.13	cd	3924.38	ab	1948.75	c
NZnO(100%D)	26320	ab	2277.19	b	39731.25	a	24903.13	a	3034.17	c	2387.19	a
NZnO(50%D+50%F)	27580	a	2055	b	34709.38	abc	24237.5	ab	3904.69	ab	2023.13	bc
NZnO(75%D)	22804	b	1872.19	b	27278.13	cd	15539.69	cd	2815	c	1769.69	c
NZnO(50%D)	25900	ab	2024.38	b	23362.5	d	13335.63	d	3102.19	c	1893.44	c
NZnO(75%F)	28000	a	2317.5	b	37693.75	ab	18362.5	cd	3174.69	bc	2359.38	ab
NZnO(50%F)	28420	a	2511.25	ab	33993.75	abc	19028.13	bc	4055.31	a	2516.25	a
ROOTS												
Zn^2+^(100%D)	15330	abc	2275.63	a	12576.56	ab	20470	a	1950.31	a	2291.88	abc
Zn^2+^(50%D+50%F)	12950	c	1601.19	b	11315.63	abc	14427.5	cd	1196.91	d	1856.56	c
NZnO(100%D)	16520	ab	1468.13	bc	9358.13	bcd	16820	bc	1775.94	abc	2569.69	ab
NZnO(50%D+50%F)	16940	ab	1050.03	de	5270.94	de	14197.25	cd	1868.13	ab	2134.06	bc
NZnO(75%D)	15050	abc	1037.28	de	5202.84	e	17972.5	ab	1839.06	ab	2446.25	ab
NZnO(50%D)	14280	bc	798.56	e	4680.31	e	12754.75	d	1479.22	bcd	2794.69	a
NZnO(75%F)	17220	ab	1596.56	b	15113.13	a	14652.75	cd	1401.59	cd	2489.69	ab
NZnO(50%F)	17920	a	1232.03	cd	8006.56	cde	15090	bcd	1680.94	abc	2748.44	a

Values are the mean of treatments. Different letters in each column indicate a significant difference (LSD, *p* ≤ 0.05). n = 5.

Concerning the leaf micronutrients, Cu was not affected by NZnO treatments; however, the Zn^2+^ (50%D+50%F) treatment increased the concentration of Cu by 55%. The remaining microelements increased their concentration in NZnO-treated plants. In the case of Fe and Mn, the treatments promoting an increment were NZnO (50%D+50%F) and NZnO (100%D), both raising the concentration of Fe by 29% and 26% and the concentration of Mn by 43% and 35%, with respect to control. A similar effect was observed in the concentration of Mo, where the NZnO (100% D) treatment promoted the concentration of this element by 30%. For Zn, the NZnO (50% F) and NZnO (75% F) treatments elevated the accumulation of this element by 35% and 33%, with respect to control. No significant difference was found for the remaining treatments. On the other hand, the Ni concentration increased by 55% and 34% for the NZnO (100%D) and Zn^2+^ (50%D+50%F) treatments, respectively. The Si levels increased significantly in the NZnO (50%D+50%F), NZnO (75%D), NZnO (50%D), NZnO (75%F), and NZnO (50%F) treatments with increases of 194, 166, 145, 113, and 110%, respectively, compared to the control treatment.

For the concentration of micronutrients in roots, reductions in the levels of some elements were observed in most NZnO treatments. Compared with the control, Cu was reduced by 68% with the NZnO (50%F) treatment, followed by the NZnO (50%D), NZnO (75%D), NZnO (50%D+50%F), NZnO (100%D), and NZnO (75%F) treatments, which decreased the concentration of this element by 64, 61, 60, 59 and 53%, respectively. The Zn^2+^ (50%D+50%F) treatment produced a reduction of 41%. A similar result was found in the concentration of Fe, with the level of decrease as follows: Zn^2+^ (50%D+50%F)> NZnO (75%F)> NZnO (50%F)> NZnO (50%D)> NZnO (50%D+50%F)> NZnO (75%D), with an Fe decrease compared to the control of 50, 43, 38, 34, 27 and 13%, respectively. The same effect was found for the concentration of Mn, where the NZnO treatments produced reductions from 25 to 55%. A decrease of 23–34% for Mo was obtained compared to the control.

Decreases in Zn root concentration were also found, mainly in the NZnO (50%F), NZnO (75%F), and NZnO (50%D) treatments, with reductions of 64, 52, and 41%, respectively. However, in the NZnO (75%D) treatment, an increase of 170% was observed. The concentration of Ni increased 86% compared with the control in the plants treated with NZnO (75%D). In contrast, in the NZnO (50%D+50%F) and Zn^2+^ (50%D+50%F) treatments, Ni was reduced by 60% and 41%, respectively. The concentration of Si in the roots was not affected by the NZnO treatments (Table 3).

**Table 3:** Concentration of micronutrients (mg kg^−1^ DW) in leaves and roots of lettuce.

TREATMENT	Cu		Fe		Mn		Mo		Zn		Ni		Si	
LEAVES														
Zn^2+^(100%D)	27.06	bcd	795	bc	125.78	c	5.81	bc	101.97	bc	26.53	cde	67.78	d
Zn^2+^(50%D+50%F)	41.94	a	901.03	ab	140.94	bc	6.69	ab	105.91	abc	35.59	ab	78.66	d
NZnO(100%D)	30.88	abc	1008.47	a	170.47	ab	7.56	a	131.88	ab	41.31	a	97.94	d
NZnO(50%D+50%F)	34.94	ab	1028.06	a	180.56	a	5.72	cd	130.34	ab	21.72	e	199.84	a
NZnO(75%D)	20.84	cd	797.63	bc	134.25	bc	4.84	d	89.75	c	31.5	bc	180.72	ab
NZnO(50%D)	24.75	bcd	616.56	c	123.97	c	4.94	cd	86.69	c	24.63	de	166.69	bc
NZnO(75%F)	20.53	cd	926.44	ab	118.94	c	5.06	cd	136.25	a	29.28	bcd	144.63	c
NZnO(50%F)	18.06	d	844.47	ab	151.44	abc	5.38	cd	138.34	a	29.19	bcd	142.41	c
ROOTS														
Zn^2+^(100%D)	39.65	a	1150.94	b	540.63	a	11.34	a	92.94	b	39.06	c	162.69	a
Zn^2+^(50%D+50%F)	23.4	b	577.06	f	319.91	cd	10.53	a	36.28	c	16.16	e	169.28	a
NZnO(100%D)	16.23	c	1409.69	a	463.03	ab	11	a	94	b	51.88	b	185.31	a
NZnO(50%D+50%F)	15.58	c	838.94	d	295.59	d	7.66	b	49.63	c	23.41	de	157.94	a
NZnO(75%D)	15.53	c	996.34	c	383.81	bc	8.63	b	251.59	a	73.03	a	155.81	a
NZnO(50%D)	14.15	c	755.25	de	241	d	7.47	b	54.22	c	29.69	cd	145.16	a
NZnO(75%F)	18.38	bc	648.31	ef	402.84	bc	7.81	b	43.92	c	29.09	cd	167.03	a
NZnO(50%F)	12.6	c	702.44	e	389	bc	8.31	b	32.97	c	27.91	cd	157.28	a

Values are the mean of treatments. Different letters in each column indicate a significant difference (LSD, *p* ≤ 0.05). n = 5.

**Fig. 7:**
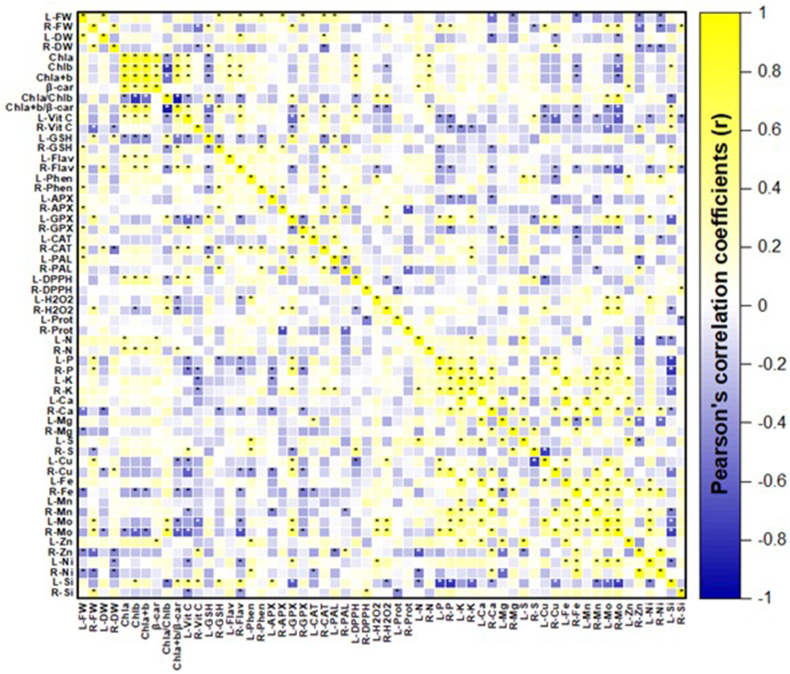
Matrix of correlations between evaluated variables of lettuce plants. L: Leaves, R: Roots, FW: Fresh weight, DW: Dry weight, Chla: Chlorophyll a, Chlb: Chlorophyll b, β-car: β-carotene, Vit C: Vitamin C, GSH: Glutathione, Flav: Flavonoids, Phen: Phenols, APX: Ascorbate peroxidase, GPX: Glutathione peroxidase, CAT: Catalase, PAL: Phenylalanine ammonium lyase, DPPH: Antioxidant capacity, H_2_O_2_: Hydrogen peroxide, Prot: Total protein.

The correlation analysis showed positive and negative relations between some of the variables studied. For example, the fresh biomass of lettuce leaves was positively related to variables of the antioxidant compounds of plants, such as phenols, flavonoids, GSH, APX, GPX, CAT, and PAL, with coefficients ranging from 0.324 to 0.414. On the other hand, a positive relationship was found between Chla and the concentration of N in leaves, with a coefficient of 0.382. Likewise, a correlation coefficient of 0.538 was found for GSH concentration and GPX activity. On the other hand, negative relationships were observed between vitamin C concentration and GSH and between vitamin C and GPX activity, with coefficients of −0.414 and −0.563, respectively. The concentration of H_2_O_2_ was positively related to the concentration of phenols and the activity of the enzymes GPX and APX, with correlation coefficients of 0.451, 0.397, and 0.360, respectively. Furthermore, the antioxidant enzymes were related to each other, where the highest coefficient was found in the activity of APX and PAL (0.726), followed by CAT and PAL (0.520), APX and CAT (0.465), GPX and PAL (0.405), and the concentration of phenols with PAL, with a correlation coefficient of 0.317. Finally, some correlations were observed between the ions, for example, the concentration of K with Fe (0.724), Cu and Mo (0.689), N and S (0.579), and K and Mn (0.501) (Fig. 7).

## 4 Discussion

By definition, a plant biostimulant is “any substance or microorganism applied to plants to enhance nutrition efficiency, abiotic stress tolerance, and/or crop quality traits, regardless of its nutrient content” [45]. Due to the positive effects of NMs on plants reported in the literature [46], it is possible to consider them biostimulants.

In general, the beneficial effects of NZnO observed in lettuce plants it's explained by the two phases of bioestimulation with NMs: The first consist in the initial contact with the cell membranes, where interactions depend of several characteristics such as size, shape, surface charges, and hydrophobicity of the NMs. The above produces damage or modifications in cell membrane, producing cascades of signaling metabolites, changes on the redox balance, membrane potential, protein translation, and modifications in gene expression. These signals can travel between cells and produce a biostimulation response [47,48]. Similar response occurs when the NMs come into contact with the organelles (e.g. chloroplasts, mitochondira or nucleus), once the NMs enter the cell through membrane pores or active mechanisms like diffusion or endocytosis, [12].

The second phase consists in the transformation (mainly ionization) of the NM core (in this case, Zn^2+^ ions) once the NMs are internalized in plant cells. The free ions in the cell cytoplasm can fulfill specific functions on several metabolic process of plants [10].

Therefore, considering the above, it is possible to explain the biostimulant impact of NZnO on growth, photosynthetic pigments, bioactive compounds, antioxidant enzyme activity, and ion concentration in lettuce plants.

### 4.1 Fresh-dry biomass of plants

The increase in fresh and dry biomass of lettuce can be explained principally by NMs promoting the photosynthetic activity of plants, increasing light absorption, accelerating the transport of energy between photosystems, and promoting the photolysis of water and the evolution of oxygen [49].

Dry weight gain may also be due to NPs increasing PSI and PSII activity, as well as the redox state of plastoquinol in the electron transport chain [50]. These increases in photosystem activity probably occur because NPs favor the overexpression of photosynthesis-related genes. Examples are *psaA* (Photosystem I P700 chlorophyll *a* apoprotein A1), *petA* (photosynthetic electron transfer A), *HSP90.1* (heat shock protein 1), and *psbA* (Photosystem II reaction center protein A) [51]. It is also possible that NPs form complexes with LHC proteins (light-harvesting complex) in the antennae of photosystems, improving light uptake [52]. In addition to participating in the light-dependent phase of photosynthesis, NMs also promote photosynthetic activity in light-independent reactions by increasing CO_2_ assimilation by boosting the activity of beta carbonic anhydrase (BCA) and Rubisco enzymes [49].

Furthermore, NZnO can increase stomatal conductance, respiratory rate, internal CO_2_ concentration, and net photosynthetic rate [53]. For example, in lettuce [54], reported that applications of 10 mg L^−1^ of NZnO increased net photosynthesis, favoring plant growth. These increases in the photosynthetic activity of plants are evidenced by the more significant accumulation of photosynthates in the leaves when applying 50–1000 mg L^−1^ of NZnO in *Brassica oleracea* [55]. On the other hand, applications of 250–500 mg L^−1^ of NZnO increase the stomatal density in the leaves, improving water balance and gas exchange, promoting the photosynthesis and respiration of plants, and resulting in greater plant growth [56].

The decrease in root biomass can be explained because NZnO causes a rearrangement of microfilaments in the epidermal cells of the elongation zones, reducing the growth of primary roots [57]. Likewise, ZnO NPs favor the generation of RNS, such as nitric oxide and peroxynitrite, causing oxidation and rearrangement of root cells and decreasing the biomass [58]. A similar result was reported by Ref. [17]; who observed that root biomass decreased when applying 300–2000 mg L^−1^ of NZnO in *Hordeum vulgare*; the authors attributed these decreases to structural changes and disorganization of root cells.

### 4.2 Photosynthetic pigments

The increases in the concentration of chlorophylls are probably related to the second stage of the biostimulation process with NMs, where the core of the material is biotransformed into ions inside the cytoplasm, specifically Zn^2+^ for the case of NZnO, which was explained in previous paragraphs [10].

In this research, increases in chlorophylls were observed in plants with foliar applications of NZnO, which can be explained by the easier internalization of the material through the leaves [59]. The NMs <2 nm can enter directly through the pores of the leaf cuticle, and NMs <20 nm can access through the stomata [60]. The NZnO used in this research has an average diameter of 16.49 nm; it follows that the access of NZnO was more efficient in the foliar application route.

Once NZnO enters cells, Zn plays a vital role in the biosynthesis of chlorophylls through the production of LHC proteins [61], increasing chlorophyll content. In addition, the same element participates in the development of chloroplasts through the expression of seven genes responsible for the membrane structure of thylakoids [62,63].

On the other hand, the decrease in the Chla/Chlb ratio can be explained because NPs promote the activity of the enzyme chlorophyll a oxygenase (EC 1.14.13.122) [64], which is responsible for synthesizing Chlb from Chla [65]. The reduction in the ratio and the increase of Chlb indicate a higher concentration of PSII against PSI, since Chlb is abundant in PSII [66]. More PSII implies a more efficient capture and use of solar radiation in the leaves. In addition to the above, Chlb fulfils essential functions, such as stabilizing LHCs and organizing thylakoidal membranes [67]. All these findings reinforce what was previously explained regarding photosynthetic efficiency and dry matter gain in plants.

The treatments did not alter the concentration of β-car in leaves, possibly due to the low production of ROS in the photosystems, since one of the functions of this pigment is the neutralization of free radicals such as O^2−^ and OH^−^ [68]. The higher Chla + b/β-car ratio shows that NPs promoted the concentration of chlorophylls compared to β-car. The latter was not altered by the treatments, possibly because the increases in β-car are more related to conditions of high solar radiation, where this compound fulfills the function of protecting chlorophylls through photoprotection and energy dissipation [69].

### 4.3 Bioactive compounds and enzymatic activity

The modifications of the plant antioxidant system with the application of NZnO were probably related to the first phase of biostimulation with NMs, as previously explained [47,48]. Specifically, the increase in bioactive compounds can be explained by the fact that NPs modify the activity of the electron transport chain in mitochondria, blocking the transfer of electrons from NADH to ubiquinone, inducing oxidative stress and increasing levels of O^2−^, H_2_O_2_, and malondialdehyde (MDA), a compound indicating lipid peroxidation of the membrane damage [50,70,71]. On the other hand, chloroplasts are one of the main ROS production sites when plants are subjected to NMs [72].

Due to the above, when exposed to NMs, plants tend to increase the production of antioxidant compounds as a defense mechanism, for example, phenolic compounds and flavonoids [73], which was evidenced in this work by finding a positive relationship between H_2_O_2_ and total phenolic compounds. In addition to this response, a positive correlation was also observed between the PAL enzyme and total phenolics, where this enzyme plays an indispensable role in the biosynthesis of these compounds [74].

On the other hand, the production of antioxidant enzymes is a mechanism to counteract the production of ROS and RNS [75,76], which was proven by the positive relationships found between H_2_O_2_ and enzymes such as APX and GPX. The higher activity of some antioxidant enzymes in this research is also partially explained because NPs can interact with these enzymes to form protein complexes, which was demonstrated by Ref. [77]. On the other hand, the increase in CAT and APX activity can be explained by the fact that NZnO induces overexpression of the *CATa*, *CATb*, and *CATc* genes, as well as *APXa* and *APXb* [20]. The overexpression of the APX gene was also reported by Ref. [17] when applying 300–2000 mg L^−1^ of NZnO in *Hordeum vulgare*.

The increase in phenolic compounds in this research coincides with [78]. They observed that applications of 160 mg L^−1^ of NZnO promoted a rise in these compounds in *Juniperus procera*. The increase in flavonoids in lettuce roots can be explained because NZnO favors the overexpression of related genes like *Solyc08g007210.3*, *Solyc08g078030.3*, and *Solyc10g078220.2* in *Solanum lycopersicum* roots [62].

The increase in the concentration of vitamin C can be explained because the synthesis of this compound in the Smirnoff-Wheeler pathway depends on the production of photoassimilates [104], which probably increased when making NZnO applications, as explained in the previous section. In turn, vitamin C contributes to neutralizing ROS in chloroplasts [105]. As in this research, an increase in vitamin C concentration was reported by Ref. [103] in tomato fruits treated with NMs. On the other hand, the decrease in GSH levels can be explained because the transformation of DHA in vitamin C depends directly of GSH in the ascorbate-glutathione cycle [106]. A similar response was reported by Ref. [107], who observed a 20% decrease in GSH concentration when applying 5–10 mg L^−1^ of NZnO in *Hordeum vulgare*. The same trend of an increase in vitamin C and a reduction in GSH was reported by Ref. [79] when applying 50–100 mg L^−1^ NZnO to *Glycine max* plants. This effect was also observed in *Moringa oleifera* seedlings when applying NZnO 2.5–10 mg L^−1^ via seed priming [28]. The relationship between vitamin C and GSH was demonstrated in this research using correlation analyses.

The increases in CAT and APX activity found in this work are similar to those reported by Ref. [78]; where applications of 80–160 mg L^−1^ of NZnO in *Juniperus procera* increased the activity of CAT and APX enzymes. Similarly, the application of 1000 mg L^−1^ NZnO to *Oryza sativa* increased the activity of CAT and other enzymes, such as superoxide dismutase (SOD) and peroxidase (POX) [53]. Similarly, PAL activity was increased in lettuce tissues with NZnO application, which has also been reported in *Capsicum annum* leaves and fruits where plants were subjected to applications of Se, Si, and Cu NPs [74].

On the other hand, the decrease in GPX activity is partially explained by the low concentration of GSH found in lettuce tissues since the above compound represents the substrate for the synthesis of GPX [42]. In addition, a reduction in GPX enzyme activity was reported in wheat and corn roots and leaves when applying NZnO 50–200 mg L^−1^ [71].

In general, NPs promote the production of antioxidant compounds to achieve cell homeostasis, keeping ROS levels in balance and mitigating damage to organelles [80]. This statement is reinforced by the positive correlations found between the compounds studied in this research.

### 4.4 Leaf-root mineral concentration

NMs alter the concentrations of macro-and micronutrients in plants to varying degrees [81]. This work found that most of the elements studied, mainly Ca, Mg, S, Fe, Mn, and Zn, resulted in increases in plants treated with NZnO. The increase in the concentration of these elements can be explained because NPs favor the production of organic acids exudated by the root, mainly oxalic, citric, lactic, and fumaric acids [82], which promote the absorption of nutrients by plants.

Another possible mechanism to explain the increase in minerals in lettuce is because NZnO promotes aquaporins through the overexpression of the *Tip1:1* and *Pip1:1* genes, which increases water and nutrient access to plant cells [83]. In addition to the production of water channels, NPs can create new pores in cell walls [84], promoting ion diffusion. Increases in mineral concentration in tissues may also be explained by NZnO promoting a greater abundance of root hairs [85].

On the other hand, it has been shown that applications of TiO_2_ and SiO_2_ NPs in plants can reduce the pH of the rhizosphere by up to 17.4% [82], while NZnO reduced the pH from 6.18 to 6.08 after just 7 days of exposure [86]. Generally, at a pH of 6–6.5, it is possible to obtain the highest availability of most of the essential nutrients by the roots, which partially can explain the increases in some elements, such as Ca, Mg, Fe, and Mn.

The increase in Ca concentration can be partially explained by the NZnO-linked overexpression of the *cation/H*^*+*^ *antiporter 18-like*, *calcium-transporting ATPase 13*, and *autoinhibited Ca*^*2+*^*-transporting ATPase 10* genes, while the higher concentration of S can be explained by overexpression of the *high affinity sulfate transporter type 1* gene [62].

Increased tissue Ca and Fe levels were reported with applications of NZnO in *Brassica chinensis* [87]. The concentration of Fe was also increased in leaves of *Spinacia oleracea* and *Coriandrum sativum* when subjected to 100 mg L^−1^ of NZnO via drench [88].

As expected, Zn was increased in plants treated with foliar application of NZnO, which is explained by the more significant absorption through the leaves [59], while in the roots, the access of NPs is carried out through other mechanisms, such as endocytosis [20].

On the other hand, some elements resulted without alterations or with specific decreases when performing NZnO applications. For example, the N concentration in the leaves and roots of lettuce plants subjected to NZnO showed no difference compared to the application of Zn^2+^. The same effect was reported by Ref. [89]; where ZnO NPs did not alter the concentration of N in ryegrass leaves, even though N was increased in NZnO-treated soils. Currently, it is unknown how NMs applications might affect the mechanisms of absorption, transport, and accumulation of N in plants.

Similarly, there are no reports on the effect of NPs on the absorption and transport of P in plants; however, the decrease in this element in lettuce plants could be related to the inhibition of the *PHT1*, *PHT2*, *PHT3*, and *PHT4* genes [108]. In addition, it is known that NPs can affect the expression of genes linked to other elements, such as *OsLCT1* and *OsNramp5*, which are related to the absorption and transport of Cd [90], as well as a low expression of the *OsLis1* and *OsLis2* genes, which are associated with the absorption of As [91]. Another possible explanation is the P immobilization, which has been reported in soils [92] and could have occurred in the substrate.

A decrease in the concentration of P in plants has been reported when applying a wide variety of NPs (CeO_2_, Fe_3_O_4_, SnO_2_, Ag, Co, and Ni), with reductions from 7 to 38% in the levels of this element in tomato leaves [93]. A similar result was reported by Ref. [94]; where Ag NPs reduced the concentration of P in the leaves by 14%, as well as a 34% decrease in *Glycine max* roots. The same effect was recorded when applying 20–200 mg L^−1^ of NZnO in *Arabidopsis thaliana* [95].

On the other hand, the increase in S and decrease in P can be partially explained by anion transport processes, through which plants maintain the balance of ionic charges [109].

Despite reductions in some minerals in lettuce leaves and roots, element concentrations were within adequate levels for this plant species from an agronomic point of view [96,97]. There was, therefore, no decrease in the nutritional quality of the plants.

The effects of NPs on the concentration of minerals in plant tissues are still poorly understood, and a trend of increase or decrease of these elements is not easily predictable since the responses are different depending on the environment, plant species, and the characteristics of the nanomaterial studied [98].

## 5 Conclusions

The results indicate that it is possible to completely replace Zn^2+^ with NZnO without affecting the vegetative growth of lettuce plants, in addition to increasing aerial biomass, bioactive compounds, and the accumulation of essential minerals, obtaining a better response when making foliar applications of NZnO between 50 and 75% of the total Zn^2+^ applied conventionally. Among the limitations of this study, we can find a lack of analysis of important variables such as those related to photosynthesis and possibly the determination of the activity of other antioxidant enzymes and concentrations of ROS, RNS and RSS, the above for a greater understanding of how lettuce plants respond to the application of NZnO.

## References

[1] Stanton C., Sanders D., Krämer U., Podar D. (2022). Zinc in plants: integrating homeostasis and biofortification. Mol. Plant.

[2] Hassan M.U., Aamer M., Chattha M.U., Haiying T., Shahzad B., Barbanti L., Nawaz M., Rasheed A., Afzal A., Liu Y., Guoqin H. (2020). The critical role of zinc in plants facing the drought stress. Agriculture.

[3] Sinclair S.A., Krämer U. (2012). The zinc homeostasis network of land plants. Biochim. Biophys. Acta, Mol. Cell Res..

[4] Hänsch R., Mendel R.R. (2009). Physiological functions of mineral micronutrients (Cu, Zn, Mn, Fe, Ni, Mo, B, Cl). Curr. Opin. Plant Biol..

[5] Noulas C., Tziouvalekas M., Karyotis T. (2018). Zinc in soils, water and food crops. J. Trace Elem. Med. Biol..

[6] Sturikova H., Krystofova O., Huska D., Adam V. (2018). Zinc, zinc nanoparticles and plants. J. Hazard Mater..

[7] Elsheery N.I., Helaly M.N., El-Hoseiny H.M., Alam-Eldein S.M. (2020). Zinc oxide and silicone nanoparticles to improve the resistance mechanism and annual productivity of salt-stressed mango trees. Agronomy.

[8] Faizan M., Hayat S., Pichtel J., Hayat S.Q., Pichel J., Faizan M., Fariduddin (2020). Effects of zinc oxide nanoparticles on crop plants: a perspective analysis. Sustain. Agric. Rev..

[9] Hossain Z., Yasmeen F., Komatsu S. (2020). Nanoparticles: synthesis, morphophysiological effects, and proteomic responses of crop plants. Int. J. Mol. Sci..

[10] Juárez-Maldonado A., Tortella G., Rubilar O., Fincheira P., Benavides-Mendoza A. (2021). Biostimulation and toxicity: the magnitude of the impact of nanomaterials in microorganisms and plants. J. Adv. Res..

[11] Rivero-Montejo S. de J., Vargas-Hernandez M., Torres-Pacheco I. (2021). Nanoparticles as novel elicitors to improve bioactive compounds in plants. Agriculture.

[12] González-Morales S., Cárdenas-Atayde P.A., Garza-Alonso C.A., Robledo-Olivo A., Benavides-Mendoza A., Fernandes F.L., Pereira C.H.W., Lima R., Ghoshal S., Santaella C. (2022). Inorganic Nanopesticides and Nanofertilizers.

[13] Juárez-Maldonado A. (2022). Impact of nanomaterials on plants: what other implications do they have?. Biocell.

[14] Sardoiwala M.N., Kaundal B., Choudhury S.R. (2018). Toxic impact of nanomaterials on microbes, plants and animals. Environ. Chem. Lett..

[15] Marmiroli M., Marmiroli N., Pagano L. (2022). Nanomaterials induced genotoxicity in plant: methods and strategies. Nanomaterials.

[16] Karami M.S., De Lima R. (2016). Nanoparticles cyto and genotoxicity in plants: mechanisms and abnormalities. Environ. Nanotechnol. Monit. Manag..

[17] Azarin K., Usatov A., Minkina T., Plotnikov A., Kasyanova A., Fedorenko A., Duplii N., Vechkanov E., Rajput V.D., Mandzhieva S., Alamri S. (2022). Effects of ZnO nanoparticles and its bulk form on growth, antioxidant defense system and expression of oxidative stress related genes in *Hordeum vulgare* L. Chemosphere.

[18] Benavides-Mendoza A., Kumar V., Kumar Sristava, Suprasanna P. (2022). Plant Nutrition and Food Security in the Era of Climate Change.

[19] El-Azeim M.M., Sherif M.A., Hussien M.S., Tantawy I.A.A., Bashandy S.O. (2020). Impacts of nano and non nanofertilizers on potato quality and productivity. Acta Ecol. Sin..

[20] Chen J., Dou R., Yang Z., You T., Gao X., Wang L. (2018). Phytotoxicity and bioaccumulation of zinc oxide nanoparticles in rice (*Oryza sativa* L.). Plant Physiol. Biochem..

[21] Salama H.S.A., Badry H.H. (2020). Effect of partial substitution of bulk urea by nanoparticle urea fertilizer on productivity and nutritive value of teosinte varieties. Agron. Res..

[22] Shams A., Abbas M. (2019). Can hydroxyapatite and boron oxide nanofertilizers substitute calcium superphosphate and boric acid for broccoli (*Brassica oleracea* var. italica) grown on A heavy clay soil?. Egypt. J. Hortic..

[23] Liu R., Zhang H., Lal R. (2016). Effects of stabilized nanoparticles of copper, zinc, manganese, and iron oxides in low concentrations on lettuce (*Lactuca sativa*) seed germination: nanotoxicants or nanonutrients?. Water, Air, Soil Pollut..

[24] Ghani M.I., Saleem S., Rather S.A., Rehmani M.S., Alamri S., Rajput V.D., Kalaji H.M., Saleem N., Sial T.A., Liu M. (2022). Foliar application of zinc oxide nanoparticles: an effective strategy to mitigate drought stress in cucumber seedling by modulating antioxidant defense system and osmolytes accumulation. Chemosphere.

[25] Secretaría de Medio Ambiente y Recursos Naturales (SEMARNAT) (2002). http://www.ordenjuridico.gob.mx/Documentos/Federal/wo69255.pdf%20(Last%20access.

[26] Steiner A.A. (1961). A universal method for preparing nutrient solutions of a certain desired composition. Plant Soil.

[27] Patil S.A., Shinde D.V., Ahn D.Y., Patil D.V., Tehare K.K., Jadhav V.V., Lee J.K., Mane R.S., Shrestha N.K., Han S.H. (2014). A simple, room temperature, solid-state synthesis route for metal oxide nanostructures. J. Mater. Chem..

[28] Garza-Alonso C.A., González-García Y., Cadenas-Pliego G., Olivares-Sáenz E., Trejo-Téllez L.I., Benavides-Mendoza A. (2021). Seed priming with ZnO nanoparticles promotes early growth and bioactive compounds of Moringa oleifera. Not. Bot. Horti Agrobot..

[29] Khan M.F., Ansari A.H., Hameedullah M., Ahmad E., Husain F.M., Zia Q., Baig U., Zaheer M.R., Alam M.M., Khan A.M., Alothman Z.A., Ahmad I., Ashraf G., Aliev G. (2016). Sol-gel synthesis of thorn-like ZnO nanoparticles endorsing mechanical stirring effect and their antimicrobial activities: potential role as nano-antibiotics. Sci. Rep..

[30] Lavand A.B., Malghe Y.S. (2018). Synthesis, characterization and visible light photocatalytic activity of carbon and iron modified ZnO. J. King Saud Univ. Sci..

[31] Nagata M., Yamashita I. (1992). Simple method for simultaneous determination of chlorophyll and carotenoids in tomato fruit. J. Jpn. Soc. Food Sci. Technol..

[32] Singleton V.L., Orthofer R., Lamuela-Raventos R.M. (1999). Analysis of total phenols and other oxidation substrates and antioxidants by means of Folin Ciocalteu reagent. Methods Enzymol..

[33] Arvouet-Grand A., Vennat B., Pourrat A., Legret P. (1994). Standardization of a propolis extract and identification of principal constituents. J. Pharm. Belg..

[34] Klein B.P., Perry A.K. (1982). Ascorbic acid and vitamin A activity in selected vegetables from different geographical areas of the United States. J. Food Sci..

[35] Xue T., Hartikainen H., Piironen V. (2001). Antioxidative and growth-promoting effect of selenium on senescing lettuce. Plant Soil.

[36] Brand-Williams W., Cuvelier M.E., Berset C. (1995). Use of a free radical method to evaluate antioxidant activity. Food Sci. Technol..

[37] Patterson B.D., MacRae E.A., Ferguson I.B. (1984). Estimation of hydrogen peroxide in plant extracts using titanium (IV). Anal. Biochem..

[38] Bradford M.M. (1976). A rapid and sensitive method for the quantitation of microgram quantities of protein utilizing the principle of protein-dye binding. Anal. Biochem..

[39] Dhindsa R.S., Plumb-Dhindsa P., Thorpe T.A. (1981). Leaf senescence: correlated with increased levels of membrane permeability and lipid peroxidation, and decreased levels of superoxide dismutase and catalase. J. Exp. Bot..

[40] Nakano Y., Asada K. (1987). Purification of ascorbate peroxidase in spinach chloroplasts; its inactivation in ascorbatedepleted medium and reactivation by monodehydroascorbate radical. Plant Cell Physiol..

[41] Sykłowska-Baranek K., Pietrosiuk A., Naliwajski M.R., Kawiak A., Jeziorek M., Wyderska S., Łojkowska E., Chinou I. (2012). Effect of l-phenylalanine on PAL activity and production of naphthoquinone pigments in suspension cultures of Arnebia euchroma (Royle) Johnst. In Vitro Cell. Dev. Biol. Plant.

[42] Flohe L., Gunzler W.A. (1984). Assays of glutathione peroxidase. Methods Enzymol..

[43] Bremner J.M., Norman A.G. (1965). Methods for Soil Analysis. Part 2. Chemical and Microbiological Properties.

[44] Sánchez-Navarro J.F., González-García Y., Benavides-Mendoza A., Morales-Díaz A.B., González-Morales S., Cadenas-Pliego G., García-Guillermo M.D.S., Juárez-Maldonado A. (2021). Silicon nanoparticles improve the shelf life and antioxidant status of lilium. Plants.

[45] du Jardin P. (2015). Plant biostimulants: definition, concept, main categories and regulation. Sci. Hortic..

[46] Méndez-López A., González-García Y., Juárez-Maldonado A., Ghorbanpour M., Muhammad Adnan S. (2022). Nano-Enabled Agrochemicals in Agriculture.

[47] Benavides-Mendoza A., González-Moscoso M., Ojeda-Barrios D.L., Fuentes-Lara L.O., Ingle A.P. (2021). Nanotechnology in Plant Growth Promotion and Protection: Recent Advances and Impacts.

[48] Juárez-Maldonado A., Ortega-Ortíz H., Morales-Díaz A.B., González-Morales S., Morelos-Moreno A., Cabrera-De la Fuente M., Sandoval-Rangel A., Cadenas-Pliego G., Benavides-Mendoza A. (2019). Nanoparticles and nanomaterials as plant biostimulants. Int. J. Mol. Sci..

[49] Kataria S., Jain M., Rastogi A., Živčák M., Brestic M., Liu S., Tripathi D.K., Kumar T.D., Ahmad P., Sharma S., Kumar D., Kishore N. (2018). Role of nanoparticles on photosynthesis: avenues and applications. Nanomaterials in Plants, Algae and Microorganisms: Concepts and Controversies.

[50] Zoufan P., Baroonian M., Zargar B. (2020). ZnO nanoparticles-induced oxidative stress in Chenopodium murale L, Zn uptake, and accumulation under hydroponic culture. Environ. Sci. Poll. Res..

[51] Tombuloglu H., Slimani Y., Tombuloglu G., Almessiere M., Baykal A. (2019). Uptake and translocation of magnetite (Fe_3_O_4_) nanoparticles and its impact on photosynthetic genes in barley (*Hordeum vulgare* L.). Chemosphere.

[52] Tombuloglu H., Slimani Y., Tombuloglu G., Alshammari T., Almessiere M., Korkmaz A.D., Baykal A., Samia A.C.S. (2020). Engineered magnetic nanoparticles enhance chlorophyll content and growth of barley through the induction of photosystem genes. Environ. Sci. Poll. Res..

[53] Faizan M., Sehar S., Rajput V.D., Faraz A., Afzal S., Minkina T., Sushkova S., Adil M.F., Yu F., Alatar A.A., Akhter F., Faisal M. (2021). Modulation of cellular redox status and antioxidant defense system after synergistic application of zinc oxide nanoparticles and salicylic acid in rice (*Oryza sativa*) plant under arsenic stress. Plants.

[54] Xu J.B., Wang Y.L., Luo X.S., Feng Y.Z. (2017). Influence of Fe3O4 nanoparticles on lettuce (*Lactuca sativa* L.) growth and soil bacterial community structure. Chin. J. Appl. Ecol..

[55] Awan S., Shahzadi K., Javad S., Tariq A., Ahmad A., Ilyas S. (2021). A preliminary study of influence of zinc oxide nanoparticles on growth parameters of Brassica oleracea var italic. J. Saudi Soc. Agr. Sci..

[56] Salehi H., De Diego N., Chehregani Rad A., Benjamin J.J., Trevisan M., Lucini L. (2021). Exogenous application of ZnO nanoparticles and ZnSO_4_ distinctly influence the metabolic response in Phaseolus vulgaris. L. *Sci. Tot. Environ.*.

[57] Wan J., Wang R., Wang R., Ju Q., Wang Y., Xu J. (2019). Comparative physiological and transcriptomic analyses reveal the toxic effects of ZnO nanoparticles on plant growth. Environ. Sci. Technol..

[58] Molnár Á., Rónavári A., Bélteky P., Szőllősi R., Valyon E., Oláh D., Rázga Z., Ördög A., Kónya Z., Kolbert Z. (2020). ZnO nanoparticles induce cell wall remodeling and modify ROS/RNS signaling in roots of Brassica seedlings. Ecotox. Environ. Saf..

[59] Ahmed B., Rizvi A., Ali K., Lee J., Zaidi A., Khan M.S., Musarrat J. (2021). Nanoparticles in the soil–plant system: a review. Environ. Chem. Lett..

[60] Huang D., Dang F., Huang Y., Chen N., Zhou D. (2022). Uptake, translocation, and transformation of silver nanoparticles in plants. Env. Sci. Nano..

[61] Wang P., Grimm B. (2021). Connecting chlorophyll metabolism with accumulation of the photosynthetic apparatus. Trends Plant Sci..

[62] Zhang J., Wang S., Song S., Xu F., Pan Y., Wang H. (2019). Transcriptomic and proteomic analyses reveal new insight into chlorophyll synthesis and chloroplast structure of maize leaves under zinc deficiency stress. J. Proteonomics.

[63] Sharma A., Patni B., Shankhdhar D., Shankhdhar S.C. (2013). Zinc - an indispensable micronutrient. Physiol. Mol. Biol. Plants.

[64] Yoshihara S., Yamamoto K., Nakajima Y., Takeda S., Kurahashi K., Tokumoto H. (2019). Absorption of zinc ions dissolved from zinc oxide nanoparticles in the tobacco callus improves plant productivity. Plant Cell Tissue Organ Cult..

[65] Tanaka R., Tanaka A. (2011). Chlorophyll cycle regulates the construction and destruction of the light-harvesting complexes. BBA-Bioenerg..

[66] Caffarri S., Tibiletti T., Jennings R., Santabarbara S. (2014). A comparison between plant photosystem I and photosystem II architecture and functioning. Curr. Protein Pept. Sci..

[67] Voitsekhovskaja O.V., Tyutereva E.V. (2015). Chlorophyll b in angiosperms: functions in photosynthesis, signaling and ontogenetic regulation. J. Plant Physiol..

[68] Alessandro S., Havaux M. (2019). Sensing β-carotene oxidation in photosystem II to master plant stress tolerance. New Phytol..

[69] Llansola-Portoles M.J., Sobotka R., Kish E., Shukla M.K., Pascal A.A., Polívka T., Robert B. (2017). Twisting a β-carotene, an adaptive trick from nature for dissipating energy during photoprotection. J. Biol. Chem..

[70] Dai Y., Wang Z., Zhao J., Xu L., Xu L., Yu X., Wei Y., Xing B. (2018). Interaction of CuO nanoparticles with plant cells: internalization, oxidative stress, electron transport chain disruption, and toxicogenomic responses. Environ. Sci. Nano..

[71] Srivastav A., Ganjewala D., Singhal R.K., Rajput V.D., Minkina T., Voloshina M., Srivastava S., Shrivastava M. (2021). Effect of ZnO nanoparticles on growth and biochemical responses of wheat and maize. Plants.

[72] Yue L., Zhao J., Yu X., Lv K., Wang Z., Xing B. (2018). Interaction of CuO nanoparticles with duckweed (*Lemna minor* L): uptake, distribution and ROS production sites. Environ. Pol..

[73] García-López J.I., Niño-Medina G., Olivares-Sáenz E., Lira-Saldivar R.H., Barriga-Castro E.D., Vázquez-Alvarado R., Rodríguez-Salinas P.A., Zavala-García F. (2019). Foliar application of zinc oxide nanoparticles and zinc sulfate boosts the content of bioactive compounds in habanero peppers. Plants.

[74] González-García Y., Cárdenas-Álvarez C., Cadenas-Pliego G., Benavides-Mendoza A., Cabrera-De la-fuente M., Sandoval-Rangel A., Valdés-Reyna J., Juárez-Maldonado A. (2021). Effect of three nanoparticles (Se, Si and Cu) on the bioactive compounds of bell pepper fruits under saline stress. Plants.

[75] Kapoor D., Singh S., Kumar V., Romero R., Prasad R., Singh J. (2019). Antioxidant enzymes regulation in plants in reference to reactive oxygen species (ROS) and reactive nitrogen species (RNS). Plant Gene.

[76] Dayem A.A., Hossain M.K., Lee S. Bin, Kim K., Saha S.K., Yang G.M., Choi H.Y., Cho S.G. (2017). The role of reactive oxygen species (ROS) in the biological activities of metallic nanoparticles. Int. J. Mol. Sci..

[77] Liu W., Worms I., Slaveykova V.I. (2020). Interaction of silver nanoparticles with antioxidant enzymes. Environ. Sci. Nano..

[78] Salih A.M., Al-Qurainy F., Khan S., Tarroum M., Nadeem M., Shaikhaldein H.O., Gaafar A.R.Z., Alfarraj N.S. (2021). Biosynthesis of zinc oxide nanoparticles using Phoenix dactylifera and their effect on biomass and phytochemical compounds in Juniperus procera. Sci. Rep..

[79] Ahmad P., Alyemeni M.N., Al-Huqail A.A., Alqahtani M.A., Wijaya L., Ashraf M., Kaya C., Bajguz A. (2020). Zinc oxide nanoparticles application alleviates arsenic (As) toxicity in soybean plants by restricting the uptake of as and modulating key biochemical attributes, antioxidant enzymes, ascorbate-glutathione cycle and glyoxalase system. Plants.

[80] Marslin G., Sheeba C.J., Franklin G. (2017). Nanoparticles alter secondary metabolism in plants via ROS burst. Front. Plant Sci..

[81] Kopittke P.M., Lombi E., Wang P., Schjoerring J.K., Husted S. (2019). Nanomaterials as fertilizers for improving plant mineral nutrition and environmental outcomes. Environ. Sci. Nano..

[82] Ghoto K., Simon M., Shen Z.J., Gao G.F., Li P.F., Li H., Zheng H.L. (2020). Physiological and root exudation response of maize seedlings to TiO_2_ and SiO_2_ nanoparticles exposure. BioNanoScience.

[83] Akdemir H. (2021). Evaluation of transcription factor and aquaporin gene expressions in response to Al_2_O_3_ and ZnO nanoparticles during barley germination. Plant Physiol. Biochem..

[84] Singh D., Kumar A., Dasgupta N., Ranjan S., Lichtfouse E. (2020). Environmental Nanotechnology Volume 3.

[85] Anderson A.J., McLean J.E., Jacobson A.R., Britt D.W. (2018). CuO and ZnO nanoparticles modify interkingdom cell signaling processes relevant to crop production. J. Agric. Food Chem..

[86] Huang H., Chen J., Liu S., Pu S. (2022). Impact of ZnO nanoparticles on soil lead bioavailability and microbial properties. Sci. Total Environ..

[87] Shen M., Liu W., Zeb A., Lian J., Wu J., Lin M. (2022). Bioaccumulation and phytotoxicity of ZnO nanoparticles in soil-grown Brassica chinensis L. and potential risks. J. Environ. Manag..

[88] Sharifan H., Moore J., Ma X. (2020). Zinc oxide (ZnO) nanoparticles elevated iron and copper contents and mitigated the bioavailability of lead and cadmium in different leafy greens. Ecotox. Environ. Saf..

[89] Aziz Y., Shah G.A., Rashid M.I. (2019). ZnO nanoparticles and zeolite influence soil nutrient availability but do not affect herbage nitrogen uptake from biogas slurry. Chemosphere.

[90] Cui J., Liu T., Li F., Yi J., Liu C., Yu H. (2017). Silica nanoparticles alleviate cadmium toxicity in rice cells: mechanisms and size effects. Environ. Pollut..

[91] Cui J., Li Y., Jin Q., Li F. (2020). Silica nanoparticles inhibit arsenic uptake into rice suspension cells: via improving pectin synthesis and the mechanical force of the cell wall. Environ. Sci. Nano..

[92] Gil-Díaz M., Diez-Pascual S., González A., Alonso J., Rodríguez-Valdés E., Gallego J.R., Lobo M.C. (2016). A nanoremediation strategy for the recovery of an As-polluted soil. Chemosphere.

[93] Vittori Antisari L., Carbone S., Gatti A., Vianello G., Nannipieri P. (2015). Uptake and translocation of metals and nutrients in tomato grown in soil polluted with metal oxide (CeO_2_, Fe_3_O_4_, SnO_2_, TiO_2_) or metallic (Ag, Co, Ni) engineered nanoparticles. Environ. Sci. Pollut. Res..

[94] Galazzi R.M., Arruda M.A.Z. (2018). Evaluation of changes in the macro and micronutrients homeostasis of transgenic and nontransgenic soybean plants after cultivation with silver nanoparticles through ionomic approaches. J. Trace Elem. Med. Biol..

[95] Nair P.M.G., Chung I.M. (2017). Regulation of morphological, molecular and nutrient status in Arabidopsis thaliana seedlings in response to ZnO nanoparticles and Zn ion exposure. Sci. Total Environ..

[96] Hochmuth G., Maynard D., Vavrina C., Hanlon E., Simonne E. (2015).

[97] Hartz T.K., Johnstone P.R., Williams E., Smith R.F. (2007). Establishing lettuce leaf nutrient optimum ranges through DRIS analysis. Hortscience.

[98] Rizwan M., Ali S., Rehman M.Z., Riaz M., Adrees M., Hussain A., Zahir Z.A., Rinklebe J. (2021). Effects of nanoparticles on trace element uptake and toxicity in plants: a review. Ecotox. Environ. Saf..

[103] Akanbi-Gada M.A., Ogunkunle C.O., Vishwakarma V., Viswanathan K., Fatoba P.O. (2019). Phytotoxicity of nano-zinc oxide to tomato plant (Solanum lycopersicum L.): Zn uptake, stress enzymes response and influence on non-enzymatic antioxidants in fruits. Environ. Technol. Innov..

[104] Suekawa M., Fujikawa Y., Esaka M., Hossain M., Munné-Bosch S., Burritt D., Diaz-Vivancos P., Fujita M., Lorence A. (2018). Ascorbic Acid Plant Growth, Dev. Stress Toler.

[105] Smirnoff N. (2018). Ascorbic acid metabolism and functions: A comparison of plants and mammals. Free Radic. Biol. Med..

[106] Pandey P., Singh J., Achary V.M.M., Mallireddy Reddy K. (2015). Redox homeostasis via gene families of ascorbate-glutathione pathway. Front. Environ. Sci..

[107] Doğaroğlu Z.G., Köleli N. (2017). TiO2 and ZnO Nanoparticles Toxicity in Barley (Hordeum vulgare L.), CLEAN – Soil. Air, Water.

[108] Mitra G., Naeem M., Ansari A., Gill S. (2017). Essential Plant Nutrients.

[109] Kohan-Baghkheirati E., Geisler-Lee J. (2015). Gene Expression, Protein Function and Pathways of Arabidopsis thaliana Responding to Silver Nanoparticles in Comparison to Silver Ions, Cold, Salt, Drought, and Heat. Nanomater.

